# Entropy-Induced Separation of Binary Semiflexible Ring Polymer Mixtures in Spherical Confinement

**DOI:** 10.3390/polym11121992

**Published:** 2019-12-02

**Authors:** Xiaolin Zhou, Fuchen Guo, Ke Li, Linli He, Linxi Zhang

**Affiliations:** 1 Department of Physics, Zhejiang University, Hangzhou 310027, Zhejiang, China; xlzhou@zju.edu.cn (X.Z.); 11836024@zju.edu.cn (F.G.); 21736027@zju.edu.cn (K.L.); 2 Department of Physics, Wenzhou University, Wenzhou 325035, Zhejiang, China

**Keywords:** semiflexible ring polymers, segregation, bending energy, depletion effects, molecular dynamics

## Abstract

Coarse-grained molecular dynamics simulations are used to investigate the conformations of binary semiflexible ring polymers (SRPs) of two different lengths confined in a hard sphere. Segregated structures of SRPs in binary mixtures are strongly dependent upon the number density of system (*ρ*), the bending energy of long SRPs (*K*_b_, long), and the chain length ratio of long to short SRPs (α). With a low *ρ* or a weak *K*_b_, long at a small ratio α, long SRPs are immersed randomly in the matrix of short SRPs. As *ρ* and bending energy of long SRPs (*K*_b_, long) are increased up to a certain value for a large ratio α, a nearly complete segregation between long and short SRPs is observed, which can be further characterized by the ratio of tangential and radial components of long SRPs velocity. These explicit segregated structures of the two components in spherical confinement are induced by a delicate competition between the entropic excluded volume (depletion) effects and bending contributions.

## 1. Introduction

Semiflexible polymers are used in a wide variety of applications as versatile materials due to their possible liquid crystalline behaviour [1,2]. Semiflexible biopolymers are also important living matter constituents [3,4], such as double-stranded DNA, actin and microtubules, which play a central role for cell structure and function [5]. Hence, understanding the interaction between semiflexible biopolymers and cell membranes becomes a key problem with many attractive aspects. Accordingly, semiflexible linear polymer solution confined in spherical vesicles can be viewed as a typical model system for cells extracted from living organisms.

Meanwhile, some functional biopolymers, such as plasmid, genome, polyose and DNA of phages, inside the host cell widely adopt ring topological conformation [6,7,8]. The ring polymer is formed by the simple operation of joining together the two ends of a linear polymer chain. Topological constraints have a dramatic effect on the properties of ring chains compared with their linear counterparts due to the decrease in the conformational degrees of freedom [9,10,11]. The most prominent examples are their different scaling behaviour [12,13,14,15,16,17,18,19,20] and rheological properties [21,22]. For isolated rings (keeping the topological constraints; i.e., bond uncrossability) discussed in our study, the size scales with the polymerization degree (N) as *D*_g_ ~ N^VF^, with *D*_g_ and *V*_F_ = 0.589 being the diameter of gyration and the Flory exponent, respectively. However, the size of the linear chain scales is *D*_g_ ~ N^0.5^. Additionally, for the strongly entangled case, ring polymers adopt crumpled globular conformations, with an effective scaling exponent showing a crossover from *V*_F_ ~ 0.4 to *V*_F_ ~ 0.33–0.36 in the limit of large *N* [16,17,18,19,20]. The ring polymer melts also exhibit self-similar dynamics, thereby yielding a power-law stress relaxation instead of the entanglement plateau, followed by exponential decay observed in entangled linear chains [23]. The rings relax stress much faster than linear polymers, and the zero-shear viscosity varies as η_0_ ~ N^1.4^, which is much weaker than the N^3.4^ behaviour of linear chains [24]. Moreover, the shapes of ring chains change from prolate, crumpled structures to planar, rigid rings with the increase of the stiffness [25].

Semiflexible linear polymers (SLPs) in the spherical confinement has been extensively studied [26,27,28,29,30,31,32,33], whether a single (long) or many (short) semiflexible linear polymers [31,32,33]. Milchev et al. [31,32] investigated the competition between the nematic order and confinement of semiflexible linear polymers in a spherical cavity at relatively large densities by using molecular dynamics (MD) simulation. This condition leads to a complicated nonuniform structure, that is bipolar orientational order versus tennis ball states. Nevertheless, much less attention has been paid to the confinement of SRPs inside the spherical container, especially considering SRP mixtures with polydispersity. The physical effects from the topological constraints and confinements play significant roles in the structure and function of individual genetic materials [34,35,36,37,38], such as chromosome shape in elongated bacterial cells [39], DNA (or DNA-actin filament mixtures) self-organization in a cell-size confinement [40], and drug delivery from spherical vesicles [41,42], etc.

Binary mixtures of ring polymers of various lengths have been considered, and the conformations of ring polymers in bidisperse blends of ring polymers have been investigated by computer simulations [43]. However, the confinement effects, as well as chain stiffness of SRPs in the binary SRP mixtures, have not been investigated yet. In this study, we focus on the binary semiflexible ring polymer (SRP) mixtures of different lengths (long and short) and chain stiffness confined in a rigid sphere. We carefully monitor the distribution and conformation characteristic of the binary mixtures. These intriguing results are compared with their linear counterparts. At the same time, we also examine the conformations of long SRPs in binary SRP mixtures by changing the chain length of short SRPs and the number density in mixtures. The article is organized as follows: In Section 2, the model and simulation details are provided. In Section 3, our results on the entropy-induced separation of binary semiflexible ring polymer mixtures in spherical confinements are given, and in Section 4, the conclusion will be presented.

## 2. Model and Method

In our simulation, a standard bead-spring model is used to model unknotted, non-concatenated ring polymer, containing *N* spherical monomers with a diameter of *σ* and a mass of *m*, interconnected by the finitely extendable nonlinear elastic (FENE) potential [44]:

(1) $$U_{FENE}(r)=-\frac{KR_{0}^{2}}{2}\ln\left[1-{\left(\frac{r}{R_{0}}\right)}^{2}\right],r<R_{0}$$

Where *r* is the distance between two neighbouring monomers. *K* = 30*k*_B_·*T*/σ^2^ is a spring constant and *R*_0_ = 1.5σ is a finite extensibility to avoid chain crossing, where *k*_B_ is the Boltzmann constant and *T* is the temperature. To prevent overlap between monomers, a shifted and cut-off Lennard-Jones (LJ) potential is used for both bonded and non-bonded pairs interactions:

(2) $$U_{LJ}(r)=\left\{ \begin{array}{cc} 4\varepsilon [{\left(\frac{\sigma }{r}\right)}^{12}-{\left(\frac{\sigma }{r}\right)}^{6}+\frac{1}{4}] & r\leq r_{c}\\ 0 & r>r_{c} \end{array}\right.$$

where *r* is distance between any two monomers and the cutoff distance is *r*_c_ = 2^1/6^σ. Additionally, the chain stiffness of polymer is controlled by a bending potential between adjacent bonds [45,46,47,48,49]:

*U*_bending_ = *K*_b_ (1 + cosθ) (3)

where θ is the angle between two consecutive bonds and *K*_b_ is the bending energy.

In our simulation, binary SRP mixtures are confined in a hard sphere with the radius of *R* = 20σ, and the schematic diagram is given in Figure S1 (see Figure S1 in Supporting Information). The sphere surface is constructed by the LJ particle with the same diameter σ and mass m with polymer monomer. The purely repulsive surface-polymer interactions are also described by Equation (2) with the repulsion strength of ε = 1.0*k*_B_·*T*. In our simulation, all quantities are reported in reduced units (*k*_B_·*T* = 1, σ = 1, *m* = 1 and τ_0_ = (*m*·σ^2^/*k*_B_·*T*)^1/2^ = 1) and are chosen to be the units of energy, length, mass, and time, respectively.

The total number density of the mixture is defined by *ρ* = (*M*_long_ × *N*_long_ + *M*_short_ × *N*_short_)/V, and long SRP number density corresponds to *ρ*_long_ = (*M*_long_ × *N*_long_)/V, where *M*_long_ and *M*_short_ are the number of long and short SRPs, *N*_long_ and *N*_short_ are the monomer number per long and short ring polymer respectively, and *V* is the volume of confined sphere. Here *N*_long_ = 50 with *M*_long_ = 10, and *M*_short_ is varied by the number density *ρ*. To prevent the deformation of SRPs, the highest density in our simulation is up to *ρ* = 0.6. Generally, *N*_short_ = 10 and *ρ* = 0.4 except special declartion. All short SRPs are regarded as semiflexible ones (even called rigid) with *K*_b,short_ = 50, and the bending energy of long SRPs *K*_b,long_ is varied 0–50, and up to 100, representing from flexible chains to semiflexible ones, and even rigid rings, respectively.

The unknotted, non-concatenated ring polymers are firstly produced by using the well-known bead-spring model of Kremer and Grest [44,50], and then these unlinked SRPs are placed randomly in the spherical confinement. SRPs which are prepared unlinked from any other SRP have to remain unlinked at any time [51]. The optimized velocity-Verlet algorithm [52] is used to integrate Newton’s equations of motion with a time step Δ*t* = 0.006τ_0_, where τ_0_ = (*m*·σ^2^/*k*_B_·*T*)^1/2^ is an intrinsic MD time unit. Then the systems are equilibrated in the NVT ensemble until the run time up to 6 × 10^7^ (i.e., 10^10^ steps), where the first run time Δ*t*_1_ = 1 × 10^6^ is used for ensuring equilibrium. Every 10^4^ steps are taken as a sample after Δ*t*_1_. The reduced temperature is T* = 1.0 in units of ε/*k*_B_ by using a Nose-Hoover thermostat [53,54]. In order to avoid the system from being locally trapped, a slow reheat-annealing simulation is applied until the final equilibrium structure is obtained. All simulations are performed by the open-source LAMMPS molecular dynamics package (Sandia National Lab, Albuquerque, NM, US) [55].

## 3. Result and Discussion

### 3.1. Segregated Structure in Binary SRPs Mixture

**Figure 1:**
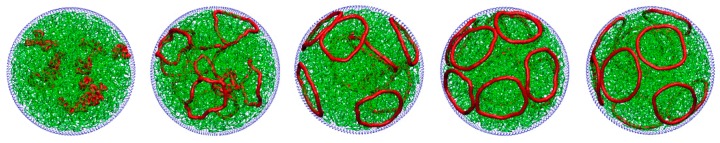
Typical snapshots of binary semiflexible ring polymers (SRP) mixtures confined in spherical confinement with different *K*_b,long_. Long SRPs are highlighted in red, and short SRPs are shown in green for clarity. Here *ρ* = 0.4, and *N*_short_ = 10.

In this section, we first focus on the mixtures of binary SRPs with different lengths (long and short) confined in a rigid sphere, where the density of long SRPs (*ρ*_long_) is much smaller than that of short SRPs. Both long and short SRPs are unknotted and unlinked mutually. The bending energy of long SRPs (*K*_b,long_) varies from 0–100, thereby representing fully-flexible to semiflexible and even rigid rings, respectively. Meanwhile, the bending energy of short SRPs is fixed at *K*_b,short_ = 50. Figure 1 shows the typical snapshots of confined binary SRP mixtures with different *K*_b,long_ at *ρ* = 0.4. When *K*_b,long_ = 0, the spherical cavity is filled with short SRPs, and the fully flexible long chains are immersed randomly in the matrix of short SRPs. However, the situation changes drastically with the increase of *K*_b,long_ from 10 to 100, and ring polymers become prolate and deformation is unfavourable, leading to that long SRPs gradually separate from the matrix of short SRPs. In particular, when *K*_b,long_ = 50, the binary SRPs mixtures have the same chain stiffness only with different lengths for long and short SRPs (i.e., *N*_long_ = 50 and *N*_short_ = 10). Almost all of long SRPs in a rigid ring conformation are attached to the surface region of sphere. This result suggests a separation between the binary long and short SRPs. The structure-segregation phenomenon becomes more evident as *K*_b,long_ = 100.

**Figure 2:**
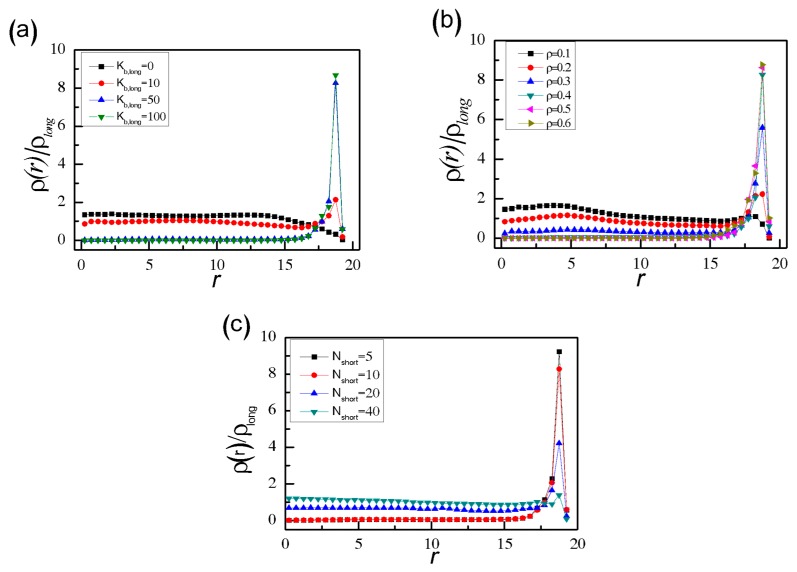
Reduced monomer density *ρ*(*r*)/*ρ*_long_ of long SRPs in spherical confinement with different *K*_b,long_ at fixed *ρ* = 0.4 and *N*_short_ = 10 (**a**), with different *ρ* at fixed *K*_b,long_ = 50 and *N*_short_ = 10 (**b**), and with different *N*_short_ at fixed *ρ* = 0.4 and *K*_b,long_ = 50 (**c**).

To check the existence of segregated structure in long and short SRPs mixtures, the reduced monomer density of long SRPs, *ρ*(*r*)/*ρ*_long_, from the sphere centre to the surface for different *K*_b,long_ is displayed in Figure 2a. The range of distance that all polymer monomers can reach is from 0–19 (see Figure S1). As expected, the density curve of flexible chains (*K*_b,long_ = 0) lacks a peak and even drops slightly when *r* is close to 19. The entropic effect of wall constraints illustrates that semiflexible short rings are more appropriate to the wall curvature than the flexible long ones [26]. When *K*_b,long_ = 10, the distribution of long SRPs is almost uniform throughout the sphere, apart from a small peak near the wall (*r* ≈ 19). As *K*_b,long_ further increases to 50 and 100, a distinct peak near the wall marks the formation of a shell of wall-attached long SRPs and a nearly complete segregation between long and short SRPs.

Then, we consider the effects of particle density *ρ* on the binary mixtures by increasing *ρ* from 0.1–0.6 at a fixed *K*_b,long_ = 50. As shown in Figure 2b, when *ρ* increases, the peak near the wall becomes sharp, and the peak height increases gradually. The binary system with low density (*ρ* = 0.1) stays homogeneous over the entire confined space, whereas the system with high density (*ρ* ≥ 0.4) rapidly develops into a nearly complete segregation of the long and short components. This result is attributed to the delicate interplay between the entropic depletion effects and packing constraints due to excluded volume and spherical confinement. In order to explore the entropic depletion attractive between the SRPs and the spherical wall in more detail, the corresponding binary mixtures in the bulk without confinement effects are shown in Figure S2. We can find that the long SRPs of binary SRPs mixtures in the bulk are partly aggregated especially for high densities such as *ρ* ≥ 0.4 (see Figure S2). In fact, there only exists depletion attraction of between the long SRPs in the bulk. However, for binary SRP mixtures in spherical confinement (see Figure 1), the depletion attraction exists not only between the long SRPs but also between long SRPs and the confining wall. Moreover, depletion attraction between long SRPs and the wall is stronger than that between the long SRPs. This strong depletion attraction can induce long SRPs to move towards the confining wall, leading a final segregated structure in binary SRP mixture in the spherical confinement. Yodh et al. [56,57] also explored the segregation behaviour of the binary colloids of different sizes trapped in a rigid vesicle, which required a sufficiently high colloid volume fraction. Depletion interactions between large sphere and wall can increase the total entropy of the system by increasing the entropy of the small spheres.

The chain length of short SRPs *N*_short_ also plays an important role in the segregation behaviour of long SRPs in binary SRP mixtures, and the reduced monomer density of long SRPs *ρ*(*r*)/*ρ*_long_ is shown in Figure 2c. For *N*_short_ = 5, and 10, the long SRPs are all located near the spherical wall. However, for *N*_short_ = 20, and 40, the binary SRPs mixture is homogenous. In fact, our system is a little similar to the binary hard-sphere mixture. In the binary hard-sphere mixture, moving two of the larger spheres towards one another does not change their interaction energy but does increase the volume accessible to the other particles, and the resulting gain in entropy reduces the free energy of the system by (3/2)α’·φ_S_·*k*_B_·*T* [57,58]. Here, α’ is the ratio of large to small radii (*R*_L_/*R*_S_), and φ_S_ is the small-sphere volume fraction. If binary hard-sphere mixtures are confined in the vesicle with different curvature, the large sphere will move in the direction of increasing curvature to minimize the small spheres’ exclude volume [59]. In this paper, the inner surface of the hard sphere is the same everywhere; that is, the long SRPs move in any direction along the inner surface of the hard sphere. Although our binary SRP system is more complicated than a binary hard-sphere system, there exists the similar entropy interaction between long SRPs and the wall, and this entropy attractive interaction is also dependent upon the chain length ratio of long to short SRPs, α = *N*_long_/*N*_short_. Our results show that the shorter the *N*_short_ is, the stronger the depletion attractive between long SRPs and the wall is. If α is less than 2.5 (i.e., *N*_short_ > 20), no segregated structure is observed for long SRPs in binary SRP mixtures.

**Figure 3:**
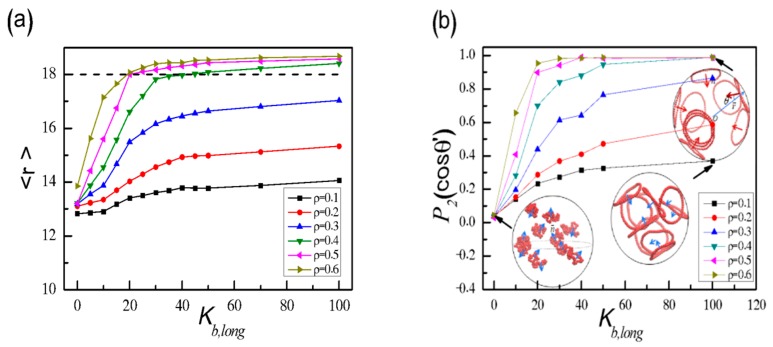
(**a**) Average distance <*r*> of long SRPs from the sphere centre to the monomers of long SRPs, and (**b**) average orientation order parameter *P*_2_(cosθ’) of long SRPs as a function of *K*_b,long_ with different *ρ*.

We also systematically examine the dependence of the separation structure for long SRPs in the binary mixtures on *K*_b,long_ and *ρ*. The average distance <*r*> from the sphere centre to the monomers of long SRPs is shown in Figure 3a. Generally, <*r*> increases with the increase in *K*_b,long_ and *ρ*. When *ρ* ≥ 0.4 and *K*_b,long_ ≥ 50, the value of <*r*> always reaches more than 18, as shown by the dotted line. This result indicates that most of the monomers of long SRPs are segregated in a wall-attached shell at the surface and short SRPs constitute the surrounding matrix in the sphere interior.

To visualize the conformation characteristic of long SRPs confined in a hard sphere, we define the average orientation order parameter *P*_2_(cosθ’) of long SRPs as follows [31,32,33]:

*P*_2_(cosθ’) = <3(cos^2^θ’ − 1)/2> (4)

where θ’ is the angle between the normal vector n of ring chains and the radius vector *r* from the centre of mass of SRPs to the sphere centre. For each long SRPs, we define the normal vector as *n* = ∑*b*_i_ × *b*_i+1_, where the sum is calculated over all pairs of consecutive bonds, with *b*_i_ and *b*_i+1_ are the corresponding bond vectors [46,47,48,49,60,61,62]. Figure 3b shows a gradual increase in *P*_2_(cosθ’) with high *ρ* and strong *K*_b,long_. As shown in the insets of Figure 3b, flexible long rings present random orientation with *P*_2_(cosθ’) close to 0, whereas *P*_2_(cosθ’) reaches 1.0 as *ρ* ≥ 0.4 and *K*_b,long_ ≥ 50. This result exhibits orientation characteristics of long SRPs. Take *ρ* = 0.4 and *K*_b,long_ = 100 for example, the average distance from the centre of the mass of SRPs to sphere centre is calculated approximately as <*r*_c_> = 17.10, which is nearly close to the perfectly ‘wall-attached’ behaviour of *r*_c_ = 17.25 as shown in Figure S3 (the gyration radius of a perfect rigid ring is <*R*_g_> = 7.95). This result demonstrates that long SRPs are pinned to the sphere surface in a planar, rigid ring conformation. Moreover, Figure S4 shows that *P*_2_(cosθ’) always keeps at about 0.55 for a low density of *ρ* = 0.2 when *N*_short_ increases from 5 to 40, however, *P*_2_(cosθ’) is close to 0.99 for *N*_short_ = 5 with a high density of *ρ* = 0.6 and decreases abruptly in the region of *N*_short_ = 10 ~ 20, and then keeps a constant value of 0.55 for *N*_short_ > 20. Therefore, the chain length of short SRPs *N*_short_ also affects seriously the average orientation of long SRPs in the spherical confinement at the high density *ρ*.

To characterize the structure transition from homogeneous to binary separation, we track the tangential and radial components of long SRPs velocity, *V*_⊥_ and *V*_//_, respectively, as follows [63]:

*V*_⊥_ = |d_δt_|sinβ/δt (5)

**Figure 4:**
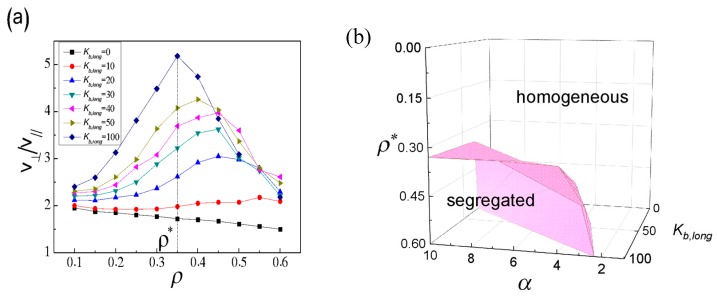
(**a**) The ratio of *V*_⊥_/*V*_//_ as a function of *ρ* for different, *K*_b,long_, and (**b**) the critical density ρ* from homogeneous to segregated structures as functions of *ρ*, *K*_b,long_ and α, corresponding to (**a**).

**Figure 5:**
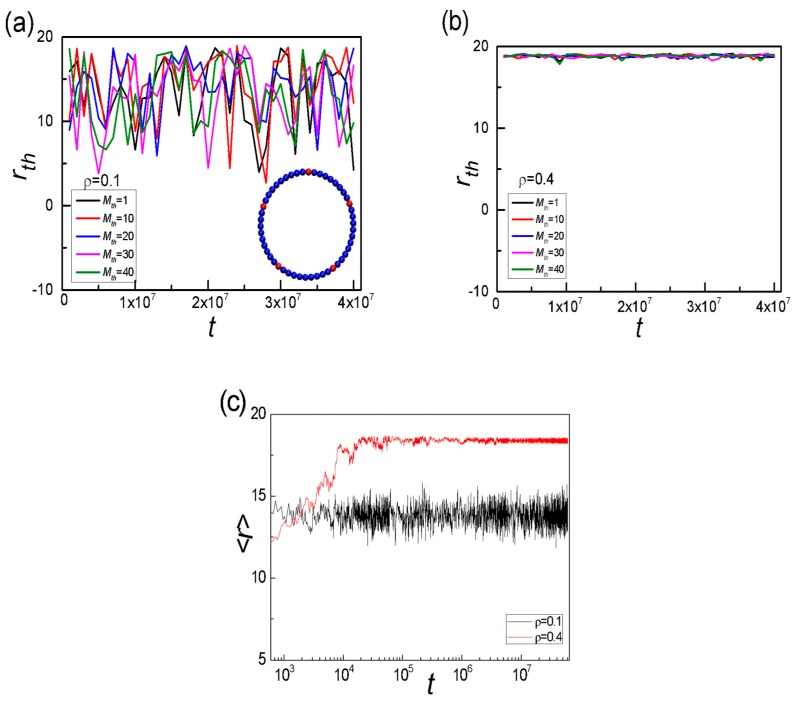
Time-dependent distance *r*_th_ from the sphere centre to the selected monomer of long SRPs for *ρ* = 0.1 (**a**), *ρ* = 0.4 (**b**), and (**c**) time-dependent average distance <*r*> from the sphere centre to the mass centre of long SRPs. Here *K*_b,long_ = 50.

*V*_//_ = |d_δt_|cosβ/δt (6)

where |d_δ*t*_| = |*r*·(*t* + δ*t*) − *r*(δ*t*)| is the displacement vector of the polymer centre of mass in the time interval δ*t*, and β is the angle between the displacement vector and normal vector of SRPs. The ratio of *V*_⊥_/*V*_//_ is plotted as *ρ* for different *K*_b,long_ in Figure 4a, here δ*t* = 2 × 10^2^. When *K*_b,long_ ≥ 20, the ratio of *V*_⊥_/*V*_//_ gradually increases, reaches a maximum, and then drops with the increase in *ρ*. Take *K*_b,long_ = 100 for example, the peak is located in ρ* = 0.35, where the perpendicular component *V*_⊥_ is dominant over the radial component *V*_//_. When *ρ* > ρ*, there are more frequent collisions of long SRPs on the sphere surface. High frequent collisions of long SRPs on the sphere surface lead to a rapid decrease of *V*_⊥_ while these collisions affect *V*_//_ slightly for high densities, see Figure S5. This result also indicates that as *ρ* > ρ*, most of long SRPs only move along the tangential direction of the sphere surface, which is in agreement with Dinsmore’s results [53]. Moreover, the critical density ρ* as a function of *K*_b,long_ and the chain length ratio of long to short SRPs, α, shown in Figure 5b, can be used to mark the structure transition from homogeneous to segregated structure. The stronger the rigidity is or the shorter the chain length of short SRPs is, the lower the critical density ρ* that can cause the phase transition is. Overall, segregated structures of long SRPs in binary mixtures depend strongly upon the number density of system (*ρ*), the bending energy of long SRPs (*K*_b,long_), and the chain length ratio of long to short SRPs (α). Except for segregated structure, the dynamic behaviour of binary SRPs mixtures is also remarkable. When *K*_b,long_ = 50, we randomly select one of the long SRPs chains and monitor the *M*_th_ = 1st, 10th, 20th, 30th and 40th monomers of the selected chain, which are marked in red in the inset of Figure 5a. The time-dependent distance *r*_th_ from sphere centre to the selected monomer of long SRPs for *ρ* = 0.1 and 0.4 are displayed in Figure 5a and b, respectively. Figure 5c further ensures that the system has reached equilibrium after Δ*t*_1_ = 1 × 10^6^. Under low density (*ρ* = 0.1), long SRPs can move throughout the inner space of sphere, while at high density (*ρ* = 0.4), the motions of the five selected monomers are trapped inside a shell of the wall with R−σ = 19. Hence, once long SRPs are separated from the short SRPs attached to the surface layer and only move along the sphere surface. Similarly, for binary hard-sphere mixtures in the vesicle confinement, the large sphere is moved to a flat wall, and the overlap volume and the free-energy loss are approximately doubled [45]. If it is the spherical confinement for binary hard-sphere mixtures, the large hard spheres move along the sphere wall, which is consistent with our results [53].

**Figure 6:**
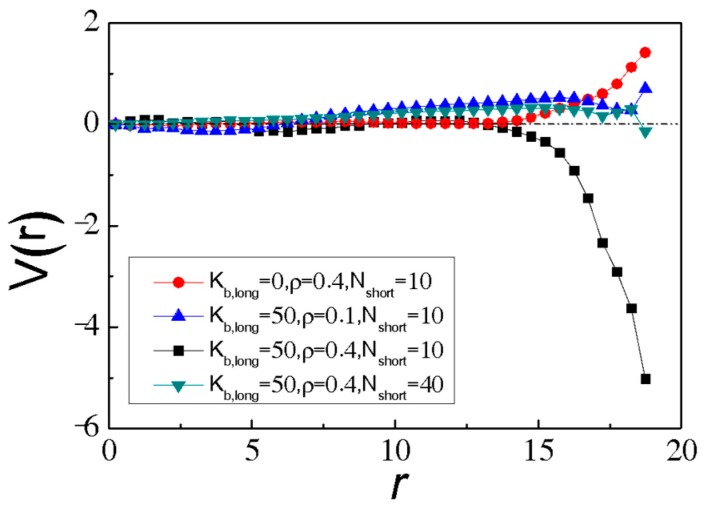
The confinement-induced long chain-wall potential mean force (PMF) for different *K*_b,long_, *ρ* and *N*_short_. Here all PMFs are shifted by *V*(*r*) = 0 as *r* = 0.

In order to explore the segregation behaviour of long SRPs in binary SRP mixture in more detail, we calculate the confinement-induced long chain-wall potential of the mean force (PMF), which represents the free energy of the system as a function of *r* relative to that of sphere centre (*r* = 0). PMF as a function of r can be calculated by *V*(*r*) = −*k*_B_·T·ln*p*(*r*) [64,65], where *p*(*r*) is the probability of finding monomers of the long chains at distance *r* during the simulation (normalized by 4πr^2^). As shown in Figure 6, for SRPs in the spherical confinement with *K*_b,long_ = 50 at a high density *ρ* = 0.4 and short *N*_short_ = 10, the PMF of system *V*(*r*) is close to zero for *r* < 13, and it drops sharply to the minimum at *r* = 18.5, which indicates that there is a strong depletion attraction between long SRPs and the wall when long SRPs are at the wall. Oppositely, for flexible long ring polymers (*K*_short_ = 0), *V*(*r*) is equal to zero in the region of *r* < 14 and positive at *r* > 14, indicating that there is a strong repulsive interaction between long flexible ring polymers and the wall when long flexible ring polymers are near the wall. Similar repulsive PMFs are found for SRP mixtures with either low density *ρ* = 0.1 or short SRPs with longer length (*N*_short_ = 40). The detailed PMFs with different bending energies (*K*_b,long_), different densities (*ρ*) and different chain lengths of short SRPs (*N*_short_) are given in Figure S6. Different depletion interactions between the long SRPs and the spherical wall lead to different conformations for long SRPs in binary SRP mixture. In fact, columnar phases of stiff ring polymers have been observed in previous simulations of stiff ring polymers in bulk [66]. In such systems, depletion interactions should not play a role, as these systems contain only single species and are not in confinement. Instead, the ordering originates from the interplay between orientational and translational entropy of the ring polymers, similar to the nematic ordering of hard rods or semiflexible polymers. However, for the spherical confinements, large depletion interactions between the long SRPs and the spherical wall can lead to the segregated structures for long SRPs in binary SRP mixture.

### 3.2. Comparison with Mixtures of Semiflexible Linear and Ring Polymers

**Figure 7:**
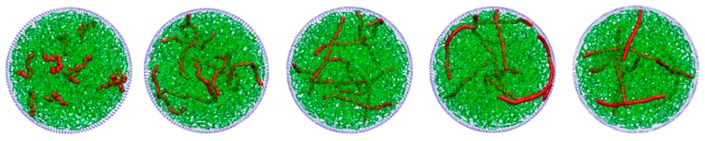
Typical snapshots of binary SLP and SRP mixtures confined in spherical confinement with different *K*_b,linear_. Long SLPs are highlighted in **red**, and short SRPs are shown in **green** for clarity. Here *ρ* = 0.4.

**Figure 8:**
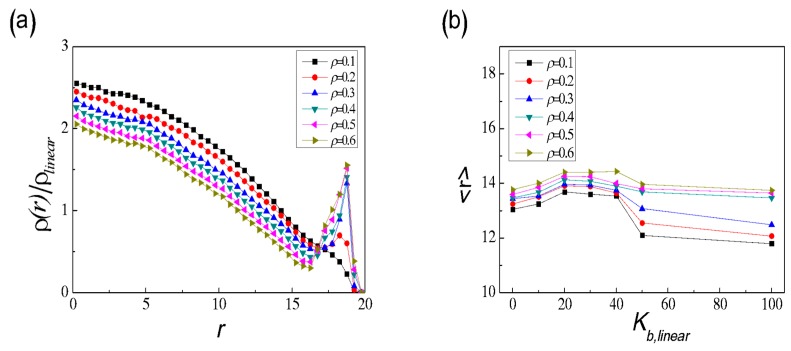
(**a**) Reduced monomer density *ρ*(*r*)/*ρ*_linear_ of long SLPs for different *ρ* with *K*_b,linear_ = 50. (**b**) Average distance <*r*> from the sphere centre to the monomers of long SLPs as a function of *K*_b,linear_ for different *ρ*.

In this section, we replace long SRPs with equal amounts of long semiflexible linear polymers (SLPs) with *N*_linear_ = 25, while keeping short SRPs content constant. The size comparison between long SRPs and long SLPs is shown in Figure S7. The typical snapshots of binary long SLP and short SRP mixtures confined in spherical confinement with different *K*_b,linear_ are shown in Figure 7. The results of *N*_linear_ = 35 is also presented in Figure S8. Long SLPs are immersed randomly in the sphere interior, while short SRPs fill up the remaining space. No obvious segregated structure is observed between long SLPs and short SRPs. By looking closely, we can find that as *K*_b,linear_ = 50, part of long SLPs are gently bent near the sphere surface to be appropriate to the wall curvature. Meanwhile, when *K*_b,linear_ = 100, most of long chains take a nearly straight conformation in the sphere interior. The corresponding reduced monomer density *ρ*(*r*)/*ρ*_long_ of long SLPs also differs significantly from their counterparts of binary SRPs, as discussed in Section 3.1. As shown in Figure 8a, the value of *ρ*(*r*)/*ρ*_long_ sharply declines from the sphere centre to the surface apart from a small peak near the wall. On the one hand, we can interpret it by the fact that long SLPs located in the sphere interior have high orientational degrees of freedom instead of the chains with most of the monomers near the sphere surface. Of course, both SRPs and SLPs lose their orientational degrees of freedom at the wall, while SRPs can attach to the wall without sacrificing of bending energy, compared with the linear ones. Therefore, the orientation entropy of long SLPs favours that many chains are close to the sphere centre. On the other hand, the density curve with a small shoulder near the wall attributes to the density oscillations “layering” induced by confinement, well known for dense fluids near hard walls [31,67,68]. Further, the average distance <*r*> from the sphere centre to the monomers of long SLPs is shown in Figure 8b. The value of <*r*> is ranged from 12.0–14.5, indicating no separation structure occurs between long SLPs and short SRPs.

**Figure 9:**
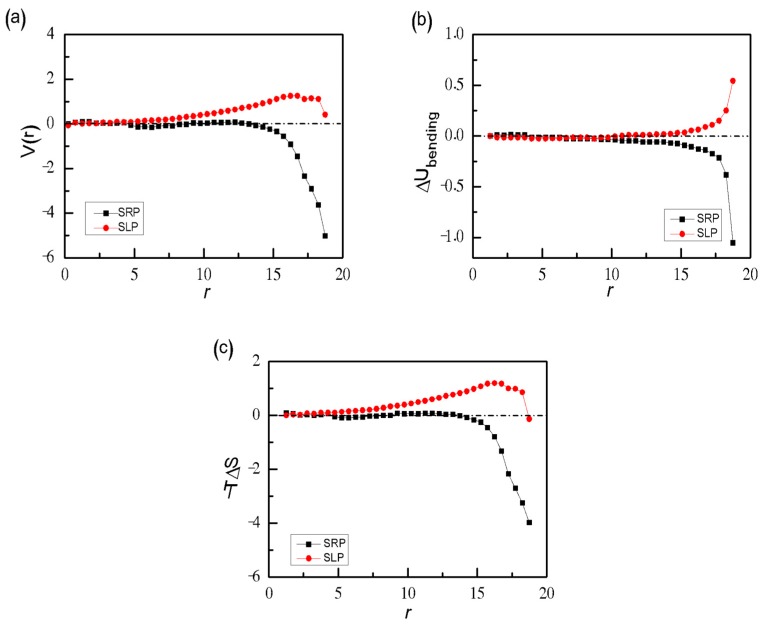
The confinement-induced long chain-wall PMF *V*(*r*) (**a**), and the energetic contribution ∆*U*_bending_ (**b**), and the entropic contribution −*T*·∆*S* (**c**), as a function of the distance *r* for SRPs and SLPs with the same bending energy *K*_b_ = 50 and the same density *ρ* = 0.4. Here *N*_long_ = 50, and *N*_linear_ = 30.

In order to quantitatively compare both ring and linear chains, we calculate the confinement-induced long chain-wall potential of the mean force (PMF), which represents the free energy of the system as a function of *r* relative to that of sphere centre (*r* = 0). As shown in Figure 9a, for confined binary SRPs mixtures, the PMF *V*(*r*) is always negative from sphere centre to sphere surface. It drops sharply to the minimum at *r* = 18.5, which indicates that there is a strong depletion attraction between long SRPs and wall. However, exactly opposite segregation behaviour is observed for confined binary SLPs and SRPs mixtures. The PMF *V*(*r*) is always positive from sphere centre to sphere surface, indicating a repulsive interaction between long SLPs and wall. As known, the free energy (*F*) consists of two constituents of energy (*U*) and entropy (*T*·*S*). The difference between SLPs and SRPs is mainly caused by different bending costs (∆*U*_bending_) near the spherical shell. We calculate the bending costs (∆*U*_bending_) for SRPs or SLPs are confined in the sphere at different r and the gained entropy (*T*·∆*S*). Here the changes of energy ∆*U*_bending_ and entropy *T*·∆*S* are defined as [61]

∆*U*_bending_ = (*U*_bending_)_confinement_ − (*U*_bending_)_bulk_ (7)

*T*·∆*S* = (*T*·*S*)_confinement_ − (*T*·*S*)_bulk_ (8)

Here (*U*_bending_)_confinement_ represents the average bending energy for polymers in spherical confinement and (*U*_bending_)_bulk_ is the average bending energy for polymers in bulk with the same density. Figure 9b shows that the bending cost ∆*U*_bending_ increases for SLPs when the SLPs move towards to the wall while it decreases abruptly for SRPs when SRPs are close to the wall. In fact, for SRPs in bulk, the ring collides with the monomers nearby with loss of bending energy. However, for SRPs near the inner surface with large *r*, due to the rigid-ring conformation, it attaches to the spherical wall without consuming bending energy. Meanwhile, as shown in Figure 9c, the gained entropy (*T*·∆*S*) due to the extra volume increases abruptly for binary SRP mixtures at *r* > 15, while there is no extra volume for binary SLP and SRP mixtures because there must be the high bending cost for long SLPs near the sphere wall. No bending costs (∆*U*_bending_ ≪ 0) and the gained entropy (*T*·∆*S* ≫ 0) for binary SRP mixture at *r* = 18.75 mean that long SPRs are willing to stay at the spherical wall, which are completely different from long SLPs in spherical confinement.

**Figure 10:**
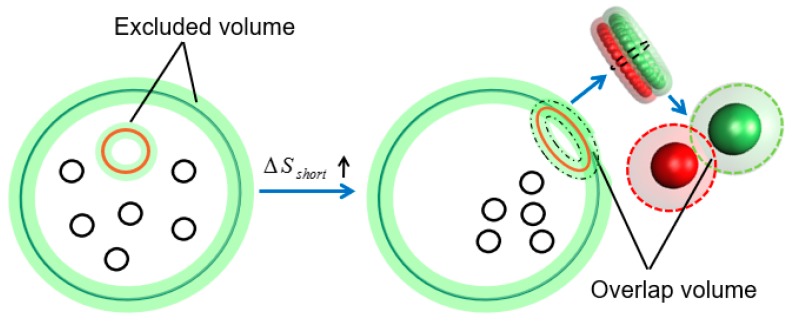
Schematic diagram and entropy-induced separation of binary SRPs mixture confined in spherical confinement.

Finally, a schematic diagram is provided to show the entropy-induced separation of binary SRPs mixtures (long and short) confined in spherical confinement. As shown in the left side of Figure 10, short SRPs (i.e., seen as small rings for semiflexible case) are excluded from the green regions within one monomer radius of the wall and of the surface of long SRPs (i.e., seen as large rings for semiflexible case). Therefore, the volume accessible to small rings, that is *V*_acc_, is the total volume minus the green regions [44,45]. When large rings move to the wall, *V*_acc_ increases by the overlapping of excluded regions and the entropy of small rings Δ*S*_short_ increases, as highlighted in the right side of Figure 10. As a result, the large rings with surface-attached chain configurations (most of their monomers in a shell near the wall) will move along the wall surface to maximize the size of the overlap region to increase the entropy of the Δ*S*_short_. This phenomenon results in an attractive “depletion” force between the large rings and wall. This entropic depletion effect drives the separation between the binary SRPs of different sizes [69,70].

## 4. Conclusions

A coarse-grained MD simulation is performed to explore the physical effects of binary semiflexible ring polymers (SRPs) of different lengths in a rigid confinement. At a high *ρ*, strong *K*_b,long_ and small *N*_short_, a nearly complete separation between the long and short SRPs occurs. The distribution and conformation characteristic of long SRPs over the course of the simulation are monitored. Long SRPs separated from short SRPs melt are attached to the sphere surface in a rigid ring conformation and only allowed to move along the sphere surface. This phenomenon can be attributed to the interplay between the entropic excluded volume (depletion) effects and bending contributions to the total free energy, i.e., no bending costs and the gained entropy due to the extra volume when the rings are at the wall. The segregation behaviour changes considerably when long SRPs are replaced by equivalent linear ones (SLPs). SLPs have the high bending cost and the related absence of the depletion force when the SLPs are at the wall. Different depletion forces lead to different spatial distributions in spherical confinement for SRPs and SLPs in binary mixtures.

## Supplementary Materials

The following are available online at https://www.mdpi.com/2073-4360/11/12/1992/s1, Figure S1: Schematic description of ring polymers confined in a spherical confinement with the excluded volume highlighted in shadow. The radius of a sphere is *R* = 20*σ*. Figure S2: Binary mixtures in the bulk without confinement effects for (a) *ρ* = 0.4 with different *K*_b,long_, and (b) *K*_b,long_ = 50 with different *ρ*. Here *N*_long_ = 50 and *N*_short_ = 10. Figure S3: Schematic description of a perfect rigid ring with *N*_ring_ = 50 attached to the sphere surface with *r*_c_ = 17.25, where *r_c_* represents the distance from the sphere center to the mass center of ring polymers. Figure S4: Average orientation order parameter *P*_2_(cosθ’) of long SRPs as a function of *N*_short_ with different *ρ*. Here *K*_b,long_ = 50, and *N*_long_ = 50. Figure S5: Tangential and radial components of long SRPs velocity *V*_⊥_ and *V*_//_ as a function of *ρ*. Here *K*_b,long_ = 50, *N*_long_ = 50, and *N*_short_ = 10. Figure S6: The potential of mean force *V*(*r*) with different *K*_b,long_ at the fixed *ρ* = 0.4 and *N*_short_ = 10(a), with different *ρ* at the fixed *K*_b,long_ = 50 and *N*_short_ = 10(b), and with different *N*_short_ at the fixed *K*_b,long_ = 50 and *ρ* = 0.4(c). Here all PMF are shifted by *V* = 0 as *r* = 0. Figure S7: The gyration radius *R*_g_ of ring polymer with *N*_ring_ = 50, and linear polymer with *N*_linear_ = 25 as a function of *K*_b_. Figure S8: Typical snapshots of binary SLP and SRP mixtures confined in spherical confinement. (a) *ρ* = 0.4 with different *K*_b,linear_, and (b) *K*_b,linear_ = 50 with different *ρ*. Here *N*_linear_ = 35 and *N*_short_ = 10.
