# Supplementary material for: Entropy-Induced Separation of Binary Semiflexible Ring Polymer Mixtures in Spherical Confinement
Source: Polymers (Basel). 2019 Dec 2;11(12):1992. doi: 10.3390/polym11121992 (PMC6960585; doi:10.3390/polym11121992)
Supplement: Supplementary file 1 [file polymers-11-01992-s001.pdf]

SUPPORTING INFORMATION

Entropy-induced separation of binary semiflexible ring polymer mixtures in spherical confinement

Xiaolin Zhou[a], Fuchen Guo[a], Ke Li[a], Linli He[b*], Linxi Zhang[a*]

[a]Department of Physics, Zhejiang University
[b]Department of Physics, Wenzhou University

*Corresponding author

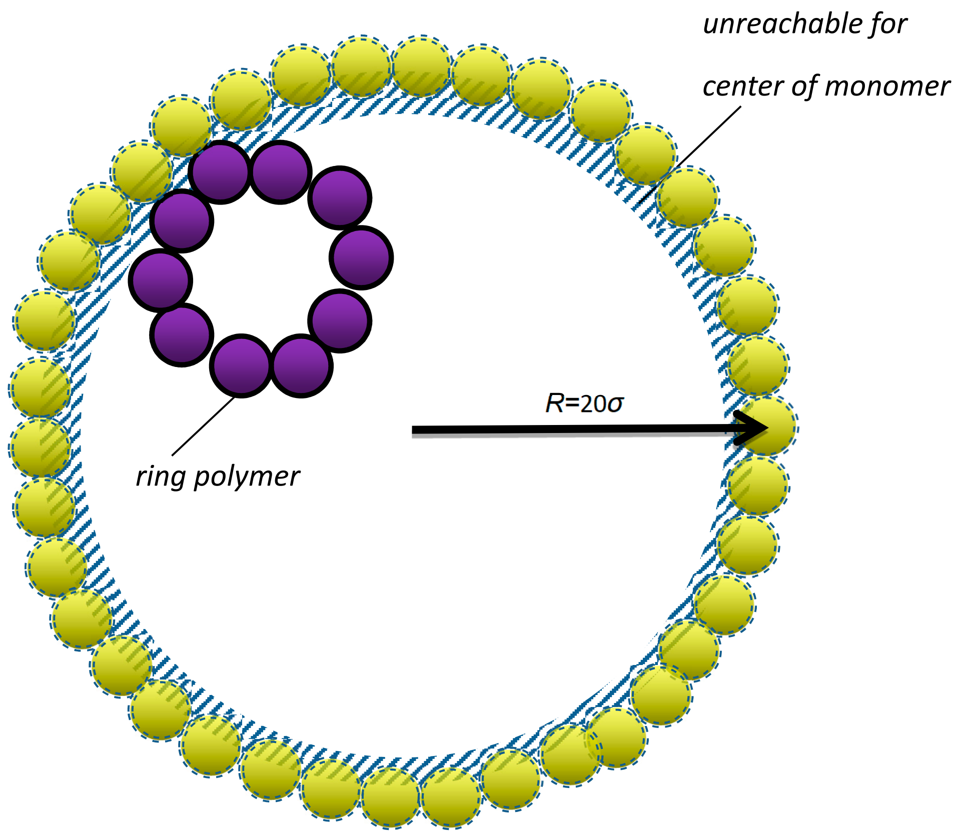

Fig. S1. Schematic description of ring polymers confined in a spherical confinement with the excluded volume highlighted in shadow. The radius of a sphere is R=20σ.

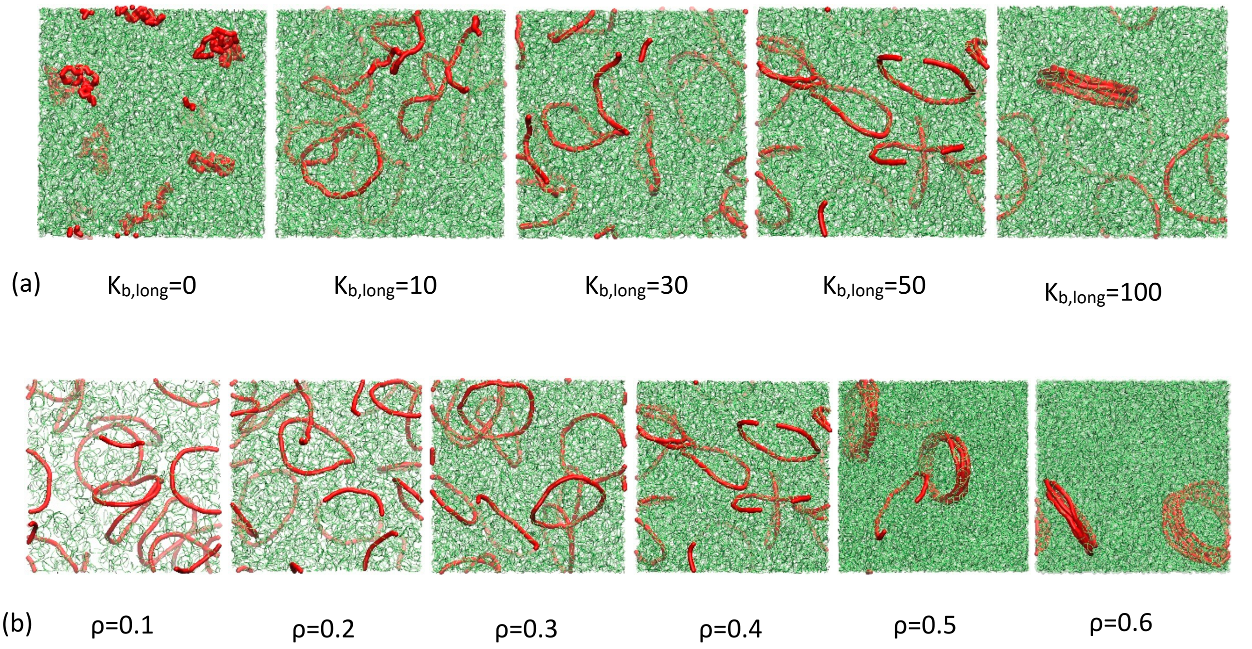

Fig. S2. Binary mixtures in the bulk without confinement effects for (a) ϱ=0.4 with different $K_{b,long}$, and (b) $K_{b,long}$=50 with different ϱ. Here N_{long}=50 and N_{short}=10.

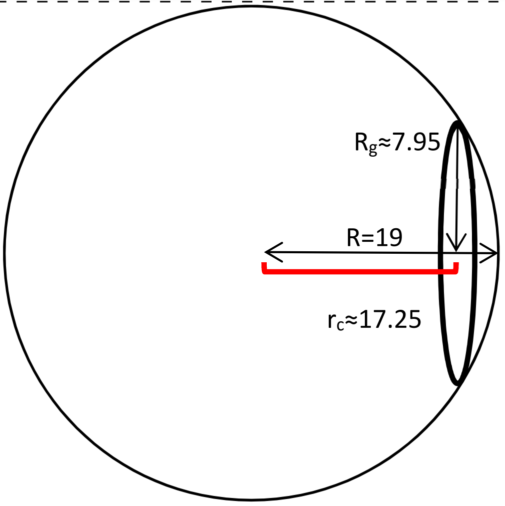

Fig. S3. Schematic description of a perfect rigid ring with N_{ring}=50 attached to the sphere surface with r_c=17.25, where r_c represents the distance from the sphere center to the mass center of ring polymers.

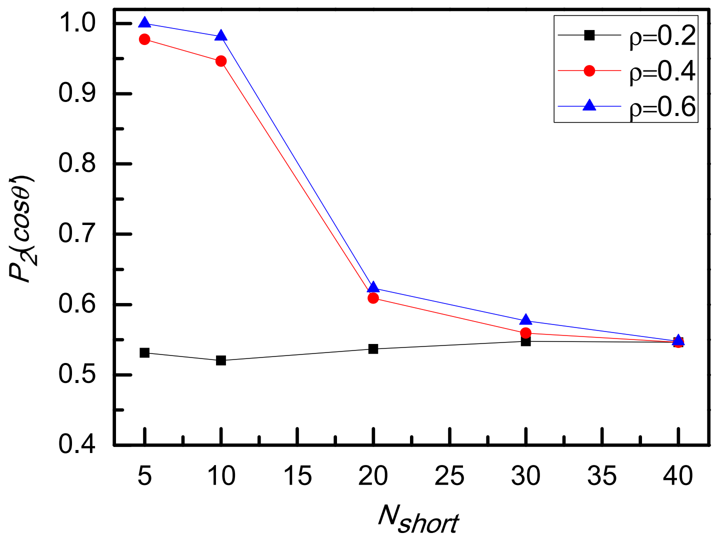

Fig. S4. Average orientation order parameter $P_2(\cos\theta')$ of long SRPs as a function of N_{short} with different ϱ. Here $K_{b,long}$=50, and N_{long}=50.

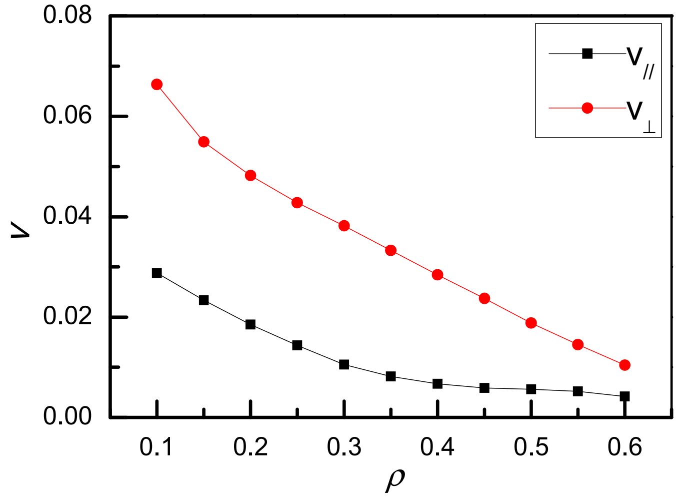

Fig. S5. Tangential and radial components of long SRPs velocity $V_{\perp}$ and $V_{//}$ as a function of ϱ. Here $K_{b,long}$=50, N_{long}=50, and N_{short}=10.

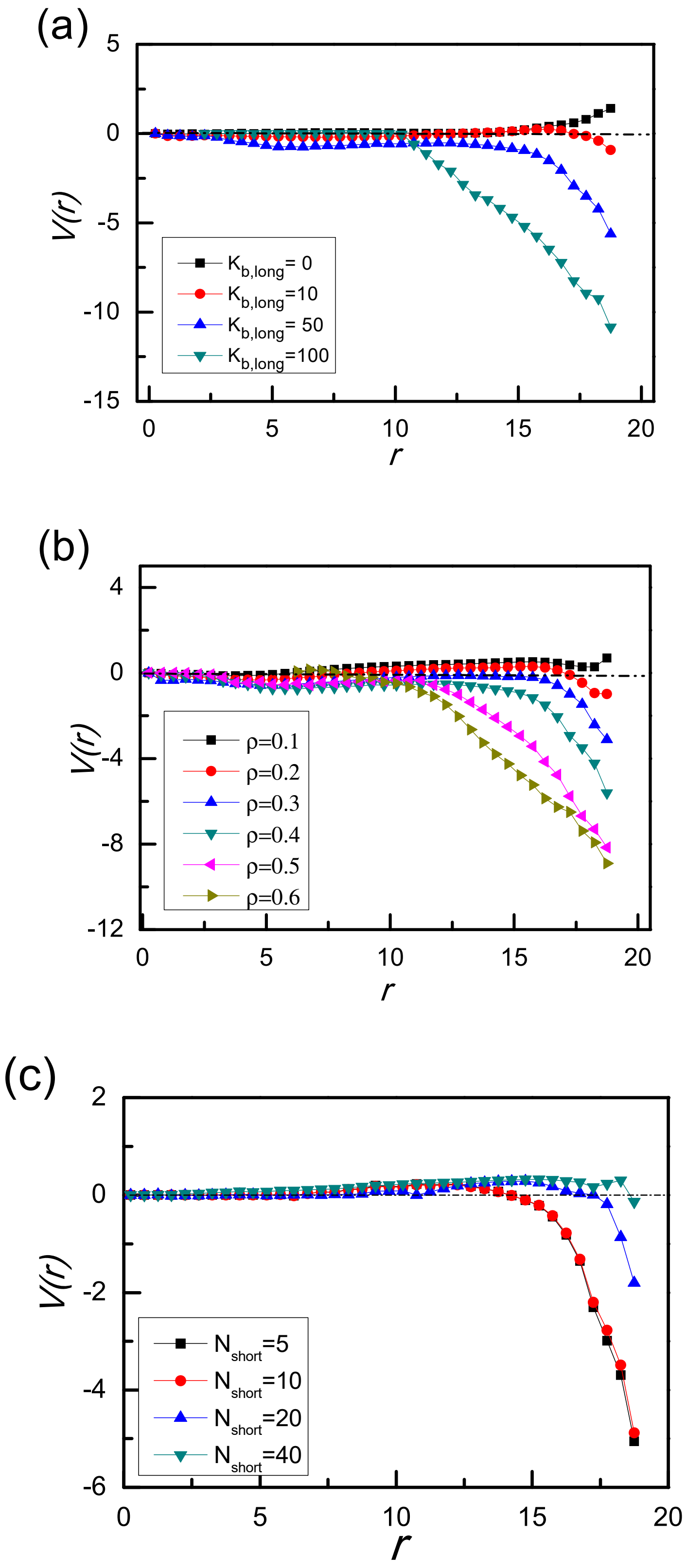

Fig. S6. The potential of mean force V(r) with different $K_{b,long}$ at the fixed ϱ=0.4 and N_{short}=10(a), with different ϱ at the fixed $K_{b,long}$=50 and N_{short}=10(b), and with different N_{short} at the fixed $K_{b,long}$=50 and ϱ=0.4(c). Here all PMF are shifted by V=0 as r=0.

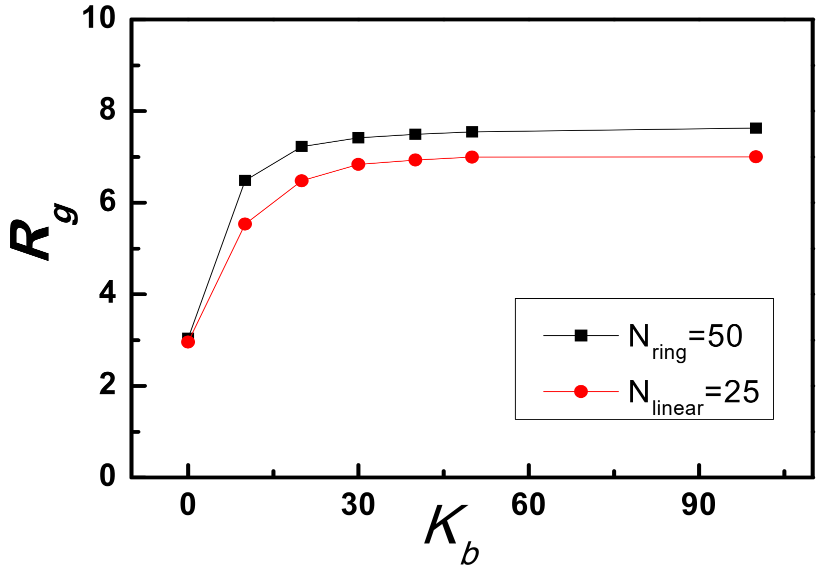

Fig. S7. The gyration radius R_g of ring polymer with N_{ring}=50, and linear polymer with N_{linear}=25 as a function of K_b.

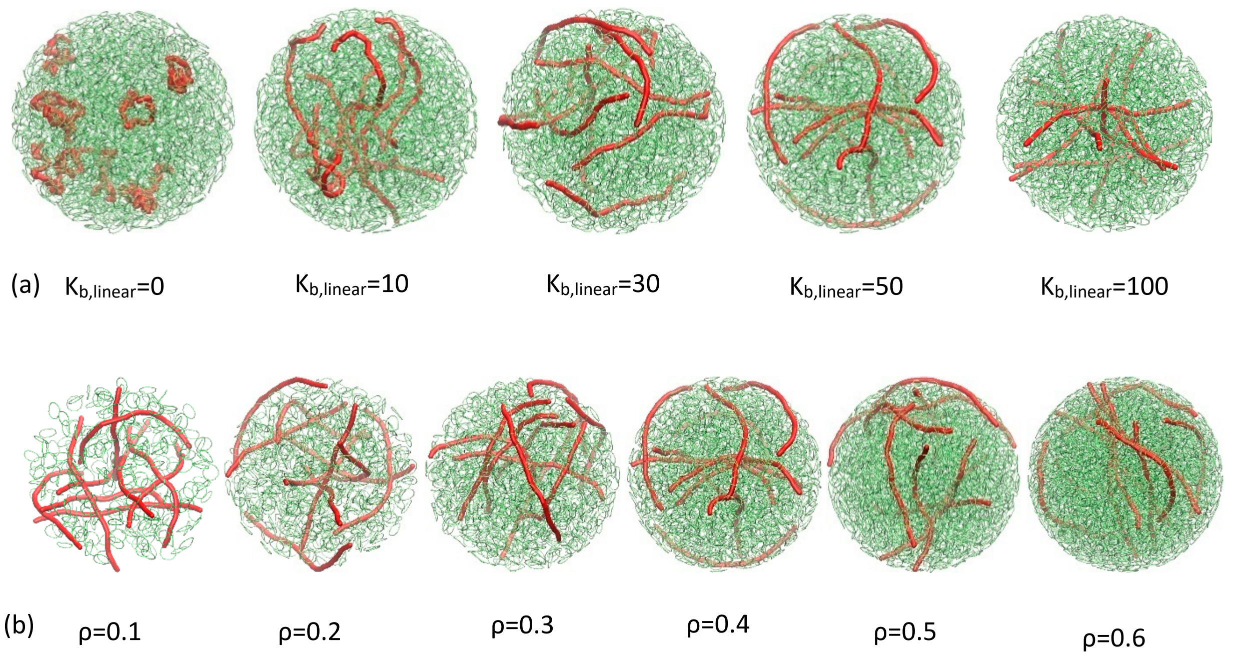

Fig. S8. Typical snapshots of binary SLP and SRP mixtures confined in spherical confinement. (a) ϱ=0.4 with different $K_{b,linear}$, and (b) $K_{b,linear}$=50 with different ϱ. Here N_{linear}=35 and N_{short}=10.
